# Supplementary material for: Clinical Utility of Ocular Assessments in Sport-Related Concussion: A Scoping Review
Source: J Funct Morphol Kinesiol. 2024 Sep 4;9(3):157. doi: 10.3390/jfmk9030157 (PMC11417888; doi:10.3390/jfmk9030157)
Supplement: Supplementary file 1 [file jfmk-09-00157-s001.zip › Supplementary File S1_Final.pdf]

Supplementary Table S1.1: Summary of Study Aims for Studies Including King-Devick (KD).

Citation	Study Aim
Galetta et al. 2011a	Determine the effect of concussion on K–D scores compared to a pre-season baseline.
Galetta et al. 2011b	'Investigate the K-D test as a potential rapid sideline screening for concussion in a cohort of boxers and mixed martial arts fighters.' (Not verbatim)
King et al. 2012	Use the K-D sideline test with the SCAT2 to see if concussions could be identified in amateur rugby league players over a representative competition period.
Leong et al. 2013	Investigate the K-D test as a screening for concussion when administered by layperson sports parents in a cohort of amateur boxers.
King et al. 2013	Use the King-Devick (KD) test and Sports Concussion Assessment Tool 2 (SCAT2) in amateur rugby union players to identify witnessed and unrecognised episodes of concussion that occurred from match participation.
Yevseyenkov et al. 2013	Evaluate the effectiveness of the King-Devick (KD) test, a screening which evaluates saccadic eye movements, to identify football players who may have sustained a concussion during play.
Galetta et al. 2015	Examine the vision-based K-D test as a complement to the current SCAT3/Child-SCAT3 measures of cognition (SAC) and balance (timed tandem gait) for sideline diagnosis of concussion.
King et al. 2015a	Determine whether the King–Devick (K–D) test used as a sideline test in junior rugby league players over 12 matches in a domestic competition season could identify witnessed and incidentally identified episodes of concussion.
King et al. 2015b	Use the King–Devick (K–D) test in senior amateur rugby union and rugby league players over a domestic competition season to see if it could identify witnessed and unwitnessed episodes of concussion that occurred from participation in competition matches over three years
Leong et al. 2015	Determine the effect of concussion on KD scores compared to pre-season baseline in collegiate level football athletes.
Vartiainen et al. 2015	Establish normative values for the K-D test using a large sample of professional male ice hockey players.
Alsalaheen et al. 2016	Purpose of this study was to (a) Describe the relationship between the K-D test and the BESS and aspects of dynamic balance as measured by the Limits of Stability (LOS) test; (b) Describe the reference values for K-D test in high school football players; (c) examine the effect of age and the history of concussions on the K-D performance; and (d) examine the relative and absolute reliability of the K-D test.
Smolyansky et al. 2016	Examine the test-retest reliability of the K-D test further, particularly in youth athletes. Unlike previous studies carried out, this study included youth athletes starting at the age of six years.
Walsh et al. 2016	Assess an "off-the-shelf" eye movement test, the King-Devick®, in those who have experienced an acute mTBI/concussion.
Dhawan et al. 2017	Examine the utility of the K-D test for real-time identification of concussion in youth hockey players.
Oberlander et al. 2017	To investigate the overall test-retest reliability of the KD among a sample of healthy adolescents.
Weise et al. 2017	Assess the repeatability of the King Devick test in the setting of mass athletic screening in high school and junior high athletes.

Supplementary Table S1.2: Summary of Study Aims for Studies Including King-Devick (KD) Continued.

Citation	Study Aim
Broglio et al. 2018	Evaluate the test-retest reliability of commonly implemented and emerging concussion assessment tools across a large nationally representative sample of student-athletes
Hecimovich et al. 2018b	(1) Identify the test–retest reliability of the K–D test in semi-elite AF players, and (2) determine the diagnostic accuracy of the K–D test in identifying SRC that occurred from game participation.
Moran and Covassin 2018a	Establish normative reference values for the KD test using a large sample of youth athletes, along with examining the baseline internal consistency of the test
Worts et al. 2018	Investigate the utility of the K-D test and VOMS in a representative adolescent sample, examine test-retest reliability, provide base rates in healthy participants and examine test performance within the context of sport participation.
Breedlove et al. 2019	(1) Assess test-retest reliability between trials, (2) test-retest reliability between years 1 and 2, and (3) reliability stratified by administration modality (spiral bound cards or tablet)
Fuller et al. 2019	Validate the KD test for identifying players with concussion in elite adult male rugby union. The primary objective was to characterise the stand-alone accuracy of the KD test in identifying concussion.
Hecimovich et al. 2018a	(1) Determine the diagnostic accuracy of the K-D Eye Tracking System (K-D/ET) test in identifying SRC that occurred from game participation, and (2) to perform a comparative analysis on saccade and blink counts for each card individually and total counts between baseline and post-injury in participants who sustained a meaningful head impact.
Naidu et al. 2018	Evaluate the utility of the K-D test in identifying sports-related concussion in professional football players.
King et al. 2020	Investigate the use of the K-D test for the sideline assessment of concussive injuries in an amateur women's rugby union team in New Zealand over two consecutive competition seasons.
Molloy et al. 2017	Determine the utility of the K–D test in identifying sports-related concussion in semi-professional rugby players.
Guzowski et al. 2017	Compare the sensitivity of an array of clinical assessment tools to SRC and, secondarily, to explore patterns of heterogeneity across athletes in clinical domains affected by SRC in order to inform recommendations about the multidimensional clinical assessment of SRC
White-Schwoch et al. 2019	Determine whether changes in performance on the FFR, BESS, and/or King-Devick (three tests of neurosensory function) are evident after one or two seasons of participation in youth tackle football.

Supplementary Table S1.3: Summary of Study Aims for Studies Including King-Devick (KD) Continued.

Citation	Study Aim
Worts et al. 2020	Determine if oculomotor fatigue (OMF) was present following a sport related concussion (SRC) in adolescents.
Elbin et al. 2019	(1) Examine baseline data for age, sex, and concussion history differences on the K–D test in a sample of non-concussed high school athletes, (2) document test–retest reliability data for the K–D test, (3) include RCIs as a method of classifying impairment, and (4) compare the sensitivity of RCIs to the previously published absolute change and MDC methods
Hecimovich et al. 2022	Determine the diagnostic accuracy of the current K-D ET test in identifying SRC that occurred from game participation and to perform a comparative analysis on saccadic and fixational activity between baseline and postinjury in concussed athletes.
Corwin et al. 2023	Use sparse principal component analysis with regression modelling to determine the combination of elements from 4 commonly used clinical concussion batteries (VVE, SCAT5, K-D, and PCSI) that maximize discrimination of adolescents with and those without concussion, thereby reducing the overall number of components required to evaluate adolescents with concussion.
Le et al. 2023	Examine the sensitivity and specificity of the K-D test at 0 to 6 hours post-concussion, 24 to 48 hours post-concussion, the beginning of an RTP protocol (asymptomatic), unrestricted RTP, and 6 months post-concussion.
Harmon et al. 2021	Assess diagnostic accuracy and reliability of sideline concussion tests in college athletes.

Supplementary Table S1.4: Summary of Study Aims for Studies Including Vestibular/Ocular Motor Screening (VOMS).

Citation	Study Aim
Mucha et al. 2014	To describe and provide initial data for the internal consistency and validity of a brief clinical screening tool for vestibular and ocular motor impairments and symptoms after sport-related concussions.
Kontos et al. 2016	The primary purpose of this study was to examine the internal consistency of the VOMS in a large sample of healthy, non-concussed collegiate athletes. The secondary purpose was to investigate the effects of patient sex and history of motion sickness, migraines, and concussions on baseline VOMS scores.
Broglio et al. 2018	To evaluate the test-retest reliability of commonly implemented and emerging concussion assessment tools across a large nationally representative sample of student-athletes
Moran and Covassin 2018b	The purpose of this study was to establish normative data and document the internal consistency and false-positive rate of the VOMS in a sample of non-concussed youth athletes.
Worts et al. 2018	To determine the test-retest reliability and rate of false-positive results of the VOMS and K-D test in a healthy athlete sample
Ferris et al. 2021a	To examine VOMS validity among collegiate student-athletes, concussed and non-concussed, from the multisite National Collegiate Athletic Association–Department of Defense Concussion Assessment, Research and Education (CARE) Consortium. A secondary aim was to utilize multidimensional machine learning pattern classifiers to deduce the additive power of the VOMS in relation to components of the Sport Concussion Assessment Tool 3 (SCAT3).
Iverson et al. 2019	The primary purpose of this study was to examine vestibular/ocular motor screening (VOMS) test performance in a sample of healthy youth ice hockey players.
Buttner et al. 2020	(1) Quantify the diagnostic accuracy of the vestibular/oculomotor screening (VOMS), and (2) Determine the recovery of vestibular and oculomotor impairments exhibited by concussed athletes compared with non-concussed athletes using the VOMS.
Knell et al. 2021	To understand the relationship between initial vestibular and ocular motor screening (VOMS) and recovery time, and the utility of VOMS to screen for protracted recovery in youth/adolescent patients with sport-related concussion (SRC).
Kontos et al. 2021	To determine the discriminative validity of individual VOMS item scores and an overall VOMS score for identifying collegiate athletes with an acute sport-related concussion (≤72 hours) from healthy controls matched by age, sex, and concussion history.
Kontos et al. 2020	The primary purpose of this study was to examine the reliability of the VOMS across a 6-month period in healthy, uninjured US Army Special Operations Command (USASOC) personnel.
Elbin et al. 2022	To develop clinical cutoffs using change scores for the VOMS individual items and an overall VOMS change score that identified concussion in adolescent athletes
Ferris et al. 2022	To investigate four hypothesis; (1) that a shortened VOMs would have equal discriminative ability to detect an acute SRC); (2) that incorporating either the current or modified VOMs into the SCAT would improve SCAT performance; (3) that incorporating the pretest symptoms into VOMs total does not improve diagnostic accuracy; (4) that using a large dataset would make it possible to make clinically meaningful integer cutoff scores for the VOMs'.
Ferris et al. 2021b	To analyze VOMS, SCAT3, and ImPACT to evaluate the test-retest reliability of consecutive-year preseason baseline assessments to directly compare the diagnostic utility of these tools when incorporating baseline assessments versus using postinjury data alone to identify acute SRC.
Moran et al. 2023	The purpose of this study was to conduct a preliminary investigation into the long-term internal consistency and inter-item relationship of VOMS symptom provocation scores, as well as test-retest reliability in a sample of child soccer athletes.
Anderson et al. 2024	Document baseline VOMS total and change scores and to document the internal consistency of the VOMS in paediatric soccer athletes

Supplementary Table S1.5: Summary of Study Aims for Studies Including Near-Point of Convergence (NPC).

Citation	Study Aim
Pearce et al. 2015	To test the reliability of repeated near point of convergence (NPC) measurements in a sample of athletes after an SRC; compare the symptoms and cognitive impairment of athletes with normal NPC to those with CI after an SRC; and explore the relationship among age, sex, learning disability, migraine history, and CI.
Kawata et al. 2015	s study intended to examine effects of repetitive sub-concussive head impacts on ocular near point of convergence (NPC).
Kawata et al. 2016	To investigate whether repetitive sub-concussive head impacts during preseason football practice cause changes in NPC.
McDevitt et al. 2016	The purpose of this study was to investigate the usefulness of 10 assessments for detecting symptoms following a concussion, which were specifically selected because they depend on vestibular, oculomotor, or sensorimotor processing
DuPrey et al. 2017	To assess the relationship between CI and recovery after SRC at the initial office visit.
Aloosh et al. 2020	Our objective was to determine the one year test-retest reliability of vision tests for concussion'. (Not verbatim)
Zonner et al. 2018	To examine the NPC response to recurring sub-concussive head impacts in a single high school football season through a series of repeated measurements.
Worts et al. 2020	To determine if oculomotor fatigue (OMF) was present following a sport-related concussion (SRC) in adolescents.
Heick et al. 2021	The purpose of this study was to determine whether four different methods of measuring NPC yielded different estimates
De Rossi, 2022	Identify normative near point of convergence (NPC) data for healthy high-school-aged athletes (13-19 years old) and determine the percentage of individuals with NPC scores that fall outside the currently accepted clinical cutoff value of 5 cm.
Kalbfell et al. 2023	To investigate the hypothesis that chronic cannabis use would mitigate elevations of NPC, S100B, and NfL levels after 20 acute headings, whereas non-cannabis users would show significant elevations in NPC, S100B, and NfL.' (Not verbatim)
Zuidema et al. 2023	To examine time-course changes in clinical (near point of convergence [NPC]) and brain-injury blood biomarkers (glial fibrillary acidic protein [GFAP], ubiquitin C-terminal hydrolase-L1 [UCH-L1], and neurofilament light [NF-L]) in adolescent football players and to test whether changes in the outcomes were associated with playing position, impact kinematics, and/or brain tissue strain.

Supplementary Table S1.6: Summary of Study Aims for Studies Including Alternative Tools and Technology.

Citation	Study Aims
Gardener et al. 2012	Examine the diagnostic efficiency of CogSport and ImPACT in athletes without baseline test data and assessed only once post-injury
Scherer et al. 2013	(1) Investigate the effects of 26 hours of sleep deprivation on DVA as a surrogate for aVOR function. (2) Explore the test-retest reliability of the DVAT for assessing aVOR function during an operationally relevant, 24-hour period during which time participants were rested.
Kaufman et al. 2013	Establish test-retest reliability of DVAT at head speeds ranging from 150 to 200 deg/s and GST in high school and college football players
Nelson et al. 2016	Evaluate the reliability and validity of three CNTs—ANAM, Axon Sports/Cogstate Sport, and ImPACT—in a common sample.
Brett et al. 2016	Investigate the reliability of baseline ImPACT scores beyond 2 years, as well as the stability of scores across three interval levels or groups of high school athletes, examining meaningful change at 1, 2, and 3 years between baselines
McDevitt et al. 2016	Investigate the usefulness of 10 assessments for detecting symptoms following a concussion, which were specifically selected because they depend on vestibular, oculomotor, or sensorimotor processing.
Tsushima et al. 2016	Evaluate the 2-year test–retest reliability of the Immediate Post-concussion Assessment and Cognitive Testing (ImPACT) neuropsychological battery, and clarified the need for biennial updated baseline testing of high school athletes.
Patterson et al. 2017	Investigate the effects of physical exertion on DVAT and to determine the reliability of DVAT in collegiate athletes as a first step in defining the role of DVAT in the sideline concussion assessment battery.
Sufrinko et al. 2017	Determine which acute vestibular, ocular motor, neurocognitive, and symptom impairments predicted SRC recovery
Broglio et al. 2018	Evaluate the test-retest reliability of commonly implemented and emerging concussion assessment tools across a large nationally representative sample of student-athletes.
Aloosh et al. 2020	Examine one-year test-retest reliability of ten vision tests in Canadian athletes over one year period of time
Cochrane et al. 2019	Complete a robust examination of visuo-oculomotor function through an eye tracking– equipped rotary chair system to determine which, if any, oculomotor functions are impaired after concussion and the test-retest reliability of those measures.
Sundaram et al. 2019	(1) Evaluate reliability of pre-practice EYE-SYNC® goggles scores; (2) evaluate reliability of post-practice EYE-SYNC® goggles scores; (3) assess the effect of exercise on change in mean pre and post-practice scores; and (4) compare pre-practice EYE-SYNC® goggles scores to the nonportable desktop-based scores; the existing system available for assessing DVS
Howell et al. 2018	Determine the test–retest correlation of an objective eye-tracking device among a sample of uninjured paediatric and adolescent athletes tested twice
Master et al. 2020	Determine whether differences in quantitative PLR metrics could serve as an objective physiologic biomarker for adolescent SRC
Feller et al. 2021	Identify ET metrics that most accurately distinguished athletes with and without concussion.
Sneigreva et al. 2021	Establish whether eye tracking technology is a reliable tool for assessing sports-related concussions in youth and adult athletes partaking in contact and collision team sports.
Ferris et al. 2021b	Analyze VOMS, SCAT3, and ImPACT to evaluate the test-retest reliability of consecutive-year preseason baseline assessments to directly compare the diagnostic utility of these tools when incorporating baseline assessments versus using postinjury data alone to identify acute SRC.
Storey et al. 2022	Evaluate the discriminatory ability of saccades and gaze stability testing at different repetition increments to determine the optimal threshold level of repetitions for differentiating adolescent athletes with and without concussion.
Harmon et al. 2021	Assess diagnostic accuracy and reliability of sideline concussion tests in college athletes
