# Supplementary material for: Clinical Utility of Ocular Assessments in Sport-Related Concussion: A Scoping Review
Source: J Funct Morphol Kinesiol. 2024 Sep 4;9(3):157. doi: 10.3390/jfmk9030157 (PMC11417888; doi:10.3390/jfmk9030157)
Supplement: Supplementary file 1 [file jfmk-09-00157-s001.zip › Supplementary File S2_Final.pdf]

Supplementary Table S2.1: Summary Extraction of King-Devick (KD) Studies.

Citation	Intra-Class Correlations (ICCs)	Classification	Reference Standard Met?
Galetta et al. 2011a	**Reliability Within-Session and Post-Fight:**		
	Pre-fight test retest (ICC = 0.97; 95% CI = 0.90–1.00),	Excellent	✓
	No SRC pre-post-fight (ICC = 0.95; 95% CI = 0.87–1.00)	Good-Excellent	✓
Leong et al. 2013	**Within Session/Pre-Post Exercise Reliability:**		
	Pre-Fight Trial 1-2 (ICC = 0.90; 95% CI = 0.84-0.97)	Good-Excellent	✓
	Pre-Post Fight (ICC = 0.96; 95% CI = 0.93-0.99)	Excellent	✓
Yevseyenkov et al. 2013	**Reliability Pre-Post Season:**		
	Baseline-Post-Season (ICC = 0.87)	Good	✓
Leong et al. 2015	**Pre-Post Season Reliability:**		
	Pre-post season (ICC = 0.95; 95% CI = 0.85-1.05)	Good-Excellent	✓
Vartiainen et al. 2015	**Within-Session Reliability:**		
	Total Score (ICC = 0.92)	Excellent,	✓
	Card 1 (ICC = 0.88)	Good	✓
	Card 2 (ICC = 0.88)	Good	✓
	Card 3 (ICC = 0.83)	Good	✓
King et al. 2015a	**Within-Session/Pre-Post Season Reliability:**		
	Baseline 1-2 (ICC = 0.92; 95% CI = 0.79 to 0.97)	Good-Excellent	✓
	Pre-Post Season (ICC = 0.92; 95% CI = 0.80 to 0.97).	Good-Excellent	✓
King et al. 2015b	**Between-Year/Pre-Post Season Reliability:**		
	Year 1 baseline 1-2 (ICC = 0.89; 95% CI 0.78 to 0.94)	Good-Excellent	✓
	Year 2 baseline 1-2 (ICC = 0.89; 95% CI = 0.78 to 0.94)	Good-Excellent	✓
	Year 3 baseline 1-2 (ICC = 0.94; 95% CI = 0.88 to 0.97)	Good-Excellent	✓
	Combined baseline 1-2 (ICC = 0.92; 95% CI = 0.88 to 0.94)	Good-Excellent	✓
	Year 1 pre-post season (ICC = 0.93; 95% CI = 0.87 to 0.97)	Good-Excellent	✓
	Year 2 pre-post season (ICC = 0.88; 95% CI = 0.76 to 0.94)	Good-Excellent	✓
	Year 3 pre-post season (ICC = 0.94; 95% CI = 0.84 to 0.96)	Good-Excellent	✓
	Combined pre-post season (ICC = 0.91; 95% CI = 0.82 to 0.94)	Good-Excellent	✓
Alsalaheen et al. 2016	**Within-Session Reliability:**		
	Baseline 1-2 (60 minute retest) (ICC = 0.89; 95% CI = 0.82-0.93)	Good-Excellent	✓
Smolyansky et al. 2016	**Within-Session Reliability:**		
	Baseline 1: ICC Trial 1-2 (ICC = 0.89; 95% CI = 0.85-0.95)	Good-Excellent	✓
	Baseline 2: ICC Trial 1-2 (ICC = 0.95; 95% CI = 0.92-0.98)	Good-Excellent	✓

	Baseline 1-2 (30 minute retest) (ICC = 0.93; 95% CI = 0.89-0.96)	Good-Excellent	☑
Oberlander et al. 2017	**Between-Session Reliability:**		
	45 day test-retest (ICC = 0.81; 95% CI = 0.73 - 0.87)	Moderate-Good	☑
Weise et al. 2017	**Within-Session Reliability:**		
	Four trials (2x2) (ICC = 0.92)	Excellent	☑
	Best score pair 1-2 (ICC = 0.94)	Excellent	☑
Broglio et al. 2018	**Between-Year Reliability:**		
	Year 1-2 (ICC = 0.74; 95% CI = 0.70-0.77).	Moderate-Good	
Hecimovich et al. 2018b	**Reliability Pre-Post Exercise and SRCs:**		
	Baseline vs SRC post-game (ICC = 0.93; 95% CI = 0.68–0.97)	Moderate-Excellent	☑
	Baseline vs Non-SRC post game (ICC = 0.92; 95% CI = 0.52–0.96)	Moderate-Excellent	☑
Moran and Covassin 2018a	**Within-Session Reliability:**		
	Trial 1-2 Card 1 (ICC = 0.92; 95% CI = 0.91-0.94)	Excellent	☑
	Trial 1-2 Card 2 (ICC = 0.91; 95% CI = 0.89-0.93)	Good-Excellent	☑
	Trial 1-2 Card 3 (ICC = 0.91; 95% CI = 0.90-0.93)	Excellent	☑
	Trial 1-2 Total Score (ICC = 0.96; 95% CI = 0.95-0.96)	Excellent	☑
Naidu et al. 2018	**Between Year/Pre-Post Exercise Reliability:**		
	Baseline Year 1-2 (ICC = 0.88; 95% CI = 0.83 – 0.91)	Good-Excellent	☑
	Pre-Post Exercise (ICC = 0.93 ; 95% CI = 0.89-0.96).	Good-Excellent	☑
Worts et al. 2018	**Within/Intra-Exercise Reliability:**		
	Baseline to pre-practice (ICC = 0.90; 95% CI, 0.82-0.94)	Good-Excellent	☑
	Baseline to intra-training (ICC = 0.87; 95% CI = 0.76-0.93)	Good-Excellent	☑
	Pre-practice to intra-training (ICC = 0.85; 95% CI = 0.72-0.92)	Moderate-Excellent	☑
	Across all time points (ICC = 0.91; 95% CI = 0.86-0.95)	Good-Excellent	☑
Breedlove et al. 2019	**Within-Session/Between Year Reliability:**		
	Trial 1-2 (ICC = 0.89)	Good	☑
	Year 1-2 (ICC = 0.83)	Good	☑
	KD Cards Year 1-2 (ICC = 0.83)	Good	☑
	KD Digital Years 1-2 (ICC = 0.83)	Good	☑
Elbin et al. 2019	**Pre-Post Season Reliability:**		
	Pre-Post Season (ICC = 0.60; 95% CI = 0.43-0.73)	X	
King et al. 2020	**Within-Session/Pre-Post Season Reliability:**		
	2018 Baseline Trial 1-2 (ICC = 0.88; 95% CI = 0.75 to 0.94)	Good-Excellent	☑
	2019 Baseline Trail 1-2 (ICC = 0.84; 95% CI = 0.62 to 0.94)	Moderate-Excellent	☑

	Pre-Post Season 2018 (ICC = 0.79; 95% CI = 0.59 to 0.89)	Moderate-Good	✓
	Pre-Post Season 2019 (ICC = 0.82; 95% CI = 0.65 to 0.91)	Moderate-Excellent	✓
	Combined Baseline Trial 1-2 (ICC = 0.89; 95% CI = 0.82 to 0.94)	Good-Excellent	✓
	Combined Pre-Post Season (ICC = 0.83; 95% CI = 0.71 to 0.90).	Moderate-Excellent	✓
Harmon et al. 2021	**Between-Year Reliability of Controls:**		
	Baseline to Retest (Median = 392 days) (ICC = 0.71; 95% CI = 0.49 to 0.84)	Poor-Good	
**Citation**	**Pearsons Correlation**	**Classification**	**Reference Standard Met?**
Vartiainen et al. 2015	**Within-Session Reliability:**		
	Total Score (r = 0.93).	Excellent,	✓
	Card 1 (r = 0.88)	Good	✓
	Card 2 (r = 0.88)	Good	✓
	Card 3 (r = 0.88)	Good	✓
White-Schwoch et al. 2019	**Pre-Post Season Reliability:**		
	Average time per card (r = 0.70; 95% CI = 0.54-87)	Moderate	
Elbin et al. 2019	**Pre-Post Season Reliability:**		
	Pre-Post Season (r = 0.61)	Moderate	
	**Spearman's Correlation**		
King et al. 2015a	**Within-Session Reliability:**		
	Baseline 1-2 (ρ=0.86; $p < 0.0001$).	High	✓
Walsh et al. 2016	**Within-Session Reliability:**		
	$\rho = 0.92$ ($p < 0.001$)	Very High	✓
	**Agreement**		
Hecimovich et al. 2018b	**Agreement:**		
	Study duration ($\kappa = 0.94$; 95% CI = 0.85–1.00).	Almost Perfect	✓
King et al. 2015b	**Agreement of Total:**		
	Combined ($\kappa = 0.98$; 95% CI = 0.94 to 1.00).	Almost Perfect	✓
**Citation**	**Internal Consistency**	**Classification**	
Galetta et al. 2011b	**Internal Consistency of Cards:**		
	Card 1 ($\alpha = 0.70$)	Acceptable	
	Card 2 ($\alpha = 0.76$)	Acceptable	
	Card 3 ($\alpha = 0.77$)	Acceptable	
King et al. 2012	**Internal Consistency of Cards:**		
	Card 1 ($\alpha = 0.72$)	Acceptable	
	Card 2 ($\alpha = 0.78$)	Acceptable	

	Card 3 (α = 0.76)	Acceptable
King et al. 2013	**Internal consistency of Cards:**	
	Card 1 (α = 0.72)	Acceptable
	Card 2 (α = 0.78)	Acceptable
	Card 3 (α = 0.76)	Acceptable
Moran and Covassin 2018a	**Internal Consistency:**	
	Overall baseline (α = 0.92)	Excellent
	Trial 1 (α = 0.88)	Good
	Trial 2 (α = 0.91)	Excellent
	Trial 1-2 Card 1 (α = 0.92)	Excellent
	Trial 1-2 Card 2 (α = 0.91)	Excellent
	Trial 1-2 Card 3 (α = 0.91)	Excellent
	Trial 1-2 Total Score (α = 0.96)	Excellent
Worts et al. 2020	**Internal Consistency in Ocular Motor Fatigue:**	
	OMF SRC athletes (α = 0.86).	Good
	Non OMF SRC athletes (α = 0.96).	Excellent
**Citation**	**Sensitivity/Specificity**	**Classification**
King et al. 2015a	Sensitivity = 1.00; 95% CI = 0.73 to 1.00).	Good-Excellent
	Specificity = 0.85; 95% CI = 0.42 to 0.97).	Acceptable
King et al. 2015b	**Sensitivity:**	
	Year 1 (1.00; 95% CI = 0.80 to 1.00)	Good-Excellent
	Year 2 (1.00; 95% CI = 0.84 to 1.00)	Good-Excellent
	Year 3 (1.00; 95% CI = 0.62 to 1.00)	Good-Excellent
	Combined (Sensitivity = 1.00; 95% CI = 0.93 to 1.00)	Good-Excellent
	**Specificity:**	
	Year 1 (1.00; 95% CI = 0.62 to 1.00)	Good-Excellent
	Year 2 (0.92; 95% CI = 0.63 to 0.99)	Good-Excellent
	Year 3 (0.97; 95% CI = 0.80 to 0.99)	Good-Excellent
	Combined (0.94; 95% CI = 0.84 to 0.99).	Good-Excellent
Dhawan et al. 2017	Optimal 2s Cutoff (Sensitivity = 0.90)	Good-Excellent
	Optimal 2s Cutoff (Specificity = 0.91)	Good-Excellent
Molloy et al. 2017	**Diagnostic Accuracy:**	
	Sensitivity = 0.53	< Acceptable
	Specificity = 0.69	< Acceptable
Hecimovich et al. 2018b	**Diagnostic Accuracy:**	

	Sensitivity (0.98; 95% CI = 0.87–1.00)	Good-Excellent
	Specificity (0.96; 95% CI = 0.80–1.00)	Good-Excellent
Naidu et al. 2018	**Diagnostic Accuracy:**	
	Sensitivity (0.62)	< Acceptable
	Specificity (0.84)	Acceptable
Hecimovich et al. 2018a	**Sensitivity:**	
	Duration (0.17; 95% CI = 0.01-0.43)	< Acceptable
	Blinks (0.33; 95% CI = 0.06-0.73)	< Acceptable
	Saccades (0.67; 95% CI = 0.27-0.94)	< Acceptable
	**Specificity:**	
	Duration (0.88; 95% CI = 0.82-0.98)	Acceptable
	Blinks (0.44; 95% CI = 0.34-0.59)	< Acceptable
	Saccades (0.69; 95% CI = 0.54-0.79)	< Acceptable
Fuller et al. 2019	**Sensitivity:**	
	Prolonged total time (0.54; 95% CI = 0.44-0.65)	< Acceptable
	Errors (0.12; 95% CI = 0.06-0.20)	< Acceptable
	Just Off-field screening (0.60; 95% CI = 0.49-0.70).	< Acceptable
	Immediate Removal and Off-Field Screening (0.60; 95% CI = 0.52-0.68)	< Acceptable
	**Specificity:**	
	Prolonged total time (0.45; 95% CI 0.31-0.60)	< Acceptable
	Errors (0.86; 95% CI = 0.74-0.94)	Acceptable
	Just Off-field screening (0.39; 95% CI = 0.26 to 0.54)	< Acceptable
	Immediate Removal and Off-Field Screening (0.39; 95% CI = 0.26 to 0.54)	< Acceptable
Harmon et al. 2021	**Cutoff Exploration:**	
	Any time increase (Sensitivity = 0.85)	Acceptable
	Any time increase (Specificity = 0.76)	< Acceptable
	Increase > 3s (Sensitivity = 0.68)	< Acceptable
	Increase > 3s (Specificity = 0.88)	Acceptable
	Increase > 5s (Sensitivity = 0.54)	< Acceptable
	Increase > 5s (Specificity = 0.95)	Good-Excellent
Hecimovich et al. 2022	**Sensitivity:**	
	Total Saccades (0.50 ; 95% CI = 0.12-0.88)	< Acceptable
	Saccade Velocity (0.67 ; 95% CI = 0.22-0.96)	< Acceptable
	Total Fixations (0.33 ; 95% CI = 0.04-0.78)	< Acceptable

	Fixation Duration (0.50 ; 95% CI = 0.12-0.88)	< Acceptable	
	Fixation Polyarea (0.67; 95% CI = 0.22-0.96)	< Acceptable	
	Completion Time (0.40 ; 95% CI = 0.05-0.85)	< Acceptable	
	**Specificity:**		
	Total Saccades (0.43; 95% CI = 0.25-0.63)	< Acceptable	
	Saccade Velocity (0.25; 95% CI = 0.11-0.45)	< Acceptable	
	Total Fixations (0.54; 95% CI = 0.34-0.72)	< Acceptable	
	Fixation Duration (0.46 ; 95% CI = 0.28-0.66)	< Acceptable	
	Fixation Polyarea (0.57 ; 95% CI = 0.37-0.76)	< Acceptable	
	Completion Time (0.86 ; 95% CI = 0.68-0.96).	Acceptable	
Le et al. 2023	**Cutoff Exploration:**		
	2.6s faster at 0-6 hours post-SRC (Sensitivity = 0.80)	Acceptable	
	3.2s faster at 24-48 hours post-SRC (Sensitivity = 0.80)	Acceptable	
	1.5s faster at 24-48 hours (Sensitivity = 0.70)	< Acceptable	
	0.8s faster at unrestricted RTP (Sensitivity = 0.80)	Acceptable	
	1.9s faster at unrestricted RTP (Sensitivity = 0.70)	< Acceptable	
	0.4s faster at 6-month post-SRC (Sensitivity = 0.80)	Acceptable	
	1.6s faster at 6-month post-SRC (Sensitivity = 0.70)	< Acceptable	
	2.6s faster at 0-6 hours post-SRC (Specificity = 0.46)	< Acceptable	
	3.2s faster at 24-48 hours post-SRC (Specificity = 0.41)	< Acceptable	
	1.5s faster at 24-48 hours (Specificity = 0.57)	< Acceptable	
	0.8s faster at unrestricted RTP (Specificity = 0.37)	< Acceptable	
	1.9s faster at unrestricted RTP (Specificity = 0.47)	< Acceptable	
	0.4s faster at 6-month post-SRC (Specificity = 0.34)	< Acceptable	
	1.6s faster at 6-month post-SRC (Specificity = 0.44)	< Acceptable	
	No cutoff could be proposed for the start of return to play (asymptomatic)		

Citation	Receiver-Operator Curve (ROC)	Classification	Reference Standard Met?
Galetta et al. 2015	**Receiver-Operator Curve (SRCs vs Control):**		
	Change from Baseline (AUC = 0.92)	Outstanding	☑
Dhawan et al. 2017	**Receiver-Operator Curve (SRC vs Control):**		
	Optimal 2s Cutoff (AUC = 0.91)	Outstanding	☑
Guzowski et al. 2017	**Receiver-Operator Curve (SRC vs Control):**		
	24-48 Hours (AUC = 0.75)	Acceptable	
Fuller et al. 2019	**Receiver-Operator Curve (SRC vs Control):**		
	Off-field screening (AUC = 0.51; 95% CI = 0.41 to 0.61) (No optimal cutoff)	Fair	

Harmon et al. 2021	**Receiver-Operator Curve (SRC vs Control):**		
	Study Duration (AUC = 0.78; 95% CI = 0.69-0.87)	Acceptable	
Hecimovich et al. 2022	**Receiver-Operator Curve (SRC vs Control) (p > 0.05 in all instances):**		
	Total Saccades (AUC = 0.55 ; 95% CI = 0.23-0.77)	Fair	
	Saccade Velocity (AUC = 0.58 ; 95% CI = 0.29-0.77)	Fair	
	Total Fixations (AUC = 0.54 ; 95% CI = 0.22-0.75)	Fair	
	Fixation Duration (AUC = 0.43 ; 95% CI = 0.18-0.64)	Poor	
	Fixation Polyarea (AUC = 0.62 ; 95% CI = 0.28-0.82).	Fair	
Le et al. 2023	**Receiver-Operator Curve (SRC vs Control):**		
	0-6-hour post SRC (AUC = 0.72; 95% CI = 0.68-0.77)	Acceptable	
	24-48 hour post SRC (AUC= 0.70; 95% CI = 0.66-0.74)	Acceptable	
	Start of return to play (asymptomatic) (AUC = 0.51; 95% CI = 0.47-0.55) (p > 0.05)	Fair	
	Unrestricted RTP from SRC (AUC = 0.64; 95% CI = 0.61-0.68)	Fair	
	6-month post-SRC (AUC = 0.62; 95% CI = 0.57-0.66)	Fair	
	KD Digital (AUC = 0.80; 95% CI = 0.75-0.85)	Acceptable	☑
	KD Card (AUC = 0.65; 95% CI = 0.60-0.69)	Fair	
	Learning Disorder (AUC = 0.82; 95% CI = 0.72-0.92)	Excellent	☑
	No Learning Disorder (AUC = 0.72; 95% CI = 0.68-0.75)	Acceptable	

Supplementary Table S2.2: Summary Extraction of Vestibular-Ocular Motor Screening (VOMs) and Near-Point of Convergence (NPC) Studies.

Citation	Intra-Class Correlation (ICC)	Classification	Reference Standard Met?
Pearce et al. 2015	Group Range (ICC = 0.95-0.98)	Excellent	☑
	Normal Convergence (ICC = 0.92-0.97)	Excellent	☑
	Convergence Insufficiency Range (ICC = 0.78 to 0.89)	Good	☑
Zonner et al. 2018	**Intra-Rater Reliability NPC across multiple time points:**		
	NPC (ICC = 0.94; 95% CI = 0.92-0.95)	Excellent	☑
Worts et al. 2018	**Same Day (+Intra-Exercise) NPC Reliability:**		
	Total (ICC = 0.91; 95% CI = 0.85-0.95)	Good-Excellent	☑
	Baseline to Pre-Practice (ICC = 0.87 [95% CI, 0.77-0.93])	Good-Excellent	☑
	Baseline to Intra-Training (ICC = 0.76; 95% CI, 0.56-0.87)	Moderate-Good	☑
Kontos et al. 2020	**Reliability between initial and 6 month follow up:**		
	Smooth pursuits (ICC = 0.60; 95% CI = 0.42–0.73)	Poor-Moderate	
	Horizontal saccades (ICC = 0.68; 95% CI = 0.53–0.78)	Moderate-Good	
	Vertical saccades (ICC = 0.75; ; 95% CI =0.63–0.83)	Moderate-Good	☑
	Near-point of convergence (ICC = 0.72; 95% CI =0.59–0.81)	Moderate-Good	
	Near-point of convergence distance (cm) (ICC = 0.77 (0.65–0.84)	Moderate-Good	☑
	Horizontal vestibular-ocular reflex (ICC = 0.74; 95% CI =0.62–0.82)	Moderate-Good	
	Vertical vestibular-ocular reflex (ICC = 0.79; 95% CI =0.69–0.85)	Moderate-Good	☑
	Visual motion sensitivity (ICC = 0.81; 95% CI =0.72–0.87)	Moderate-Good	☑
	VOMS total (ICC = 0.76; 95% CI =0.64–0.84)	Moderate-Good	☑
Aloosh et al. 2020	**1 Year Reliability of NPC via ICC**		
	NPC (ICC = 0.47; 95% CI = 0.00-0.77),	Poor-Good	
	NPCbreaк = (ICC = 0.65; 95% CI = 0.25-0.86)	Poor-Good	
Ferris et al. 2021b	**Year 1-2 Pre-Season Reliability:**		
	VOMs Total (ICC = 0.36)	Poor	
	NPC Distance (ICC = 0.60)	Moderate	
Heick et al. 2021	**Within Session Reliability of Various Size NPCs via ICC**		
	12-point font target (ICC = 0.96; 95% CI = 0.94-0.97);	Excellent	☑
	14-point font target (ICC = 0.98; 95% CI = 0.97-0.98);	Excellent	☑
	tip of the black pen (ICC = 0.95; 95% CI = 0.92-0.96);	Excellent	☑
	Bernell Vergel 9-point font device (ICC = 0.95; 95% CI = 0.92-0.96).	Excellent	☑
Kalbfell et al. 2023	**Inter-rater reliability of NPC via ICC**		

	Study Duration (ICC = 0.93; 95% CI = 0.88–0.96)	Good-Excellent	☑
Zuidema et al. 2023	**Intra and inter-rater reliability of NPC via ICC**		
	Intra-rater (ICC = 0.94; 95%CI = 0.89-0.95)	Good-Excellent	☑
	Inter-rater (ICC = 0.90; 95% CI = 0.83-0.94).	Good-Excellent	☑
Moran et al. 2023	**Reliability of Preseason VOMs over 18 months via ICC**		
	NPC (ICC = 0.31; 95% CI = 0.20-0.60)	Poor-Moderate	
**Citation**	**Pearsons Correlations**	**Classification**	**Reference Standard Met?**
Kawata et al. 2015	**Within-Session Intra-Rater Reliability of NPC:**		
	Pre-intervention (r = 0.88)	High	☑
	0-h post intervention (r = 0.95)	Very High	☑
	24-h post intervention (r = 0.94)	Very High	☑
Kawata et al. 2016	**Intra-Rater Reliability of NPC across multiple time points:**		
	Study duration (r = 0.93)	Very High	☑
Ferris et al. 2021b	**Preseason Reliability (Year 1-2)**		
	Total VOMs (r = 0.23)	Fair	
	NPC Distance (r = 0.43)	Moderate	
**Citation**	**Kappa Agreement**		
Broglio et al. 2018	**Agreement (Year 1-2)**		
	Smooth Pursuit (κ = 0.30)	Fair	
Moran et al. 2023	**Agreement (18 months)**		
	Pretest Symptoms (κ = 0.24)	Fair	
	Convergence (κ = 0.23)	Fair	
	Horz VOR (κ = 0.17)	Slight	
	Vert VOR (κ = 0.26)	Fair	
	VMS (κ = 0.26)	Fair	
**Citation**	**Internal Consistency**	**Classification**	
Mucha et al. 2014	VOMs + NPC Distance (α = .92)	Excellent	
Kontos et al. 2016	**Within-Session (NPC cutoff = 5cm, Provocation ≥ 2):**		
	VOMs Total (α = 0.97)	Excellent	
Moran and Covassin 2018b	**Within-Session (NPC cutoff = 5cm, Provocation ≥ 2):**		
	All VOMs Subscales (α = 0.97)	Excellent	
	All Subscales + Symptoms + NPC Distance (α = 0.91)	Excellent	
Iverson et al. 2019	**VOMs Subscales (NPC cutoff = 5cm, Provocation ≥ 2):**		
	Smooth pursuits (α = 0.81)	Good	

	Horz Saccades ($\alpha = 0.59$)	Poor
	Vert saccades ($\alpha = 0.70$)	Acceptable
	NPC ($\alpha = 0.72$)	Acceptable
	Horz VOR ($\alpha = 0.62$)	Questionable
	Vert VOR ($\alpha = 0.42$)	Unacceptable
	VMS ($\alpha = 0.81$)	Good
	Total ($\alpha = 0.92$)	Excellent
	Modified Total ($\alpha = 0.90$)	Excellent
Kontos et al. 2020	**6 Months (NPC Cutoff = 5cm, Provocation ≥ 2)**	
	VOMs Initial ($\alpha = 0.99$)	Excellent
	VOMs Follow Up ($\alpha = 0.99$)	Excellent
	With inclusion of average NPC:	
	Initial (Cronbach $\alpha = 0.95$)	Excellent
	Follow Up ($\alpha = 0.96$)	Excellent
Worts et al. 2020	**SRC and Ocular-Motor Fatigue**	
	Ocular-Motor Fatigue ($\alpha = 0.97$)	Excellent
	Non-Fatigued ($\alpha = 0.99$)	Excellent
Moran et al. 2023	**Preseason VOMS**	
	Initial ($\alpha = 0.92$)	Excellent
	Follow-up ($\alpha = 0.95$)	Excellent
**Citation**	**Diagnostic Accuracy**	**Classification**
DuPrey et al. 2017	**Diagnostic Accuracy for Prolonged Recovery (Cutoff = 6cm)**	
	NPC (Sensitivity = 0.84)	Acceptable
	NPC (Specificity = 0.70)	< Acceptable
Ferris et al. 2021b	**Diagnostic Accuracy**	
	VOMs Total (Sensitivity = 0.77)	< Acceptable
	VOMs Total Preseason Change (Sensitivity = 0.58)	< Acceptable
	NPC Distance (Sensitivity = 0.46)	< Acceptable
	NPC Preseason Change (Sensitivity = 0.24)	< Acceptable
	VOMs Total (Specificity = 0.83)	Acceptable
	VOMs Total Preseason Change (Specificity = 0.92)	Good-Excellent
	NPC Distance (Specificity = 0.69)	< Acceptable
	NPC Preseason Change (Specificity = 0.92)	Good-Excellent
Elbin et al. 2022	**Diagnostic Accuracy**	

	**Sensitivity**	
	Smooth pursuits (Sensitivity = 0.23) (Cutoff = 1)	< Acceptable
	Horizontal saccades (Sensitivity = 0.40) (Cutoff = 1)	< Acceptable
	Vertical saccades (Sensitivity = 0.48) (Cutoff = 1)	< Acceptable
	NPC symptoms (Sensitivity = 0.46) (Cutoff = 1)	< Acceptable
	Horizontal VOR (Sensitivity = 0.61) (Cutoff = 1)	< Acceptable
	Vertical VOR (Sensitivity = 0.59) (Cutoff = 1)	< Acceptable
	VMS (Sensitivity = 0.68) (Cutoff = 1)	< Acceptable
	NPC distance (Sensitivity = 0.51) (Cutoff ≥ 3)	< Acceptable
	Overall VOMS change score (Sensitivity = 0.64) (Cutoff ≥ 3)	< Acceptable
	**Specificity**	
	Smooth pursuits (Specificity = 0.91) (Cutoff = 1)	Good-Excellent
	Horizontal saccades (Specificity = 0.82) (Cutoff = 1)	Acceptable
	Vertical saccades (Specificity = 0.80) (Cutoff = 1)	Acceptable
	NPC symptoms (Specificity = 0.84) (Cutoff = 1)	Acceptable
	Horizontal VOR (Specificity = 0.74) (Cutoff = 1)	< Acceptable
	Vertical VOR (Specificity = 0.78) (Cutoff = 1)	< Acceptable
	VMS (Specificity = 0.73) (Cutoff = 1)	< Acceptable
	NPC distance (Specificity = 0.74) (Cutoff ≥ 3)	< Acceptable
	Overall VOMS change score (Specificity = 0.74) (Cutoff ≥ 3)	< Acceptable
Ferris et al. 2022	**Diagnostic Accuracy**	
	**Sensitivity**	
	VOMs total (Sensitivity = 0.77)	< Acceptable
	Pretest total (Sensitivity = 0.77)	< Acceptable
	Smooth Pursuit (Sensitivity = 0.79)	< Acceptable
	Horz Sacc (Sensitivity = 0.78)	< Acceptable
	Vert Sacc (Sensitivity = 0.78)	< Acceptable
	Convergence (Sensitivity = 0.78)	< Acceptable
	NPC (Sensitivity = 0.55)	< Acceptable
	VMS (Sensitivity = 0.72)	< Acceptable
	Horz VOR (Sensitivity = 0.73)	< Acceptable
	Vert VOR (Sensitivity = 0.73)	< Acceptable
	mVOMs (Sensitivity = 0.77)	< Acceptable

	Scaled mVOMs (Sensitivity = 0.77)	< Acceptable	
	Total change score (Sensitivity = 0.78)	< Acceptable	
	VOMS Total Cutoff ≥ 4 (Sensitivity = 0.78)	< Acceptable	
	mVOMS Total Cutoff ≥ 4 (Sensitivity = 0.77)	< Acceptable	
	**Specificity:**		
	VOMs total (Specificity = 0.83)	Acceptable	
	Pretest total (Specificity = 0.84),	Acceptable	
	Smooth Pursuit (Specificity = 0.84),	Acceptable	
	Horz Sacc (Specificity = 0.82),	Acceptable	
	Vert Sacc (Specificity = 0.82),	Acceptable	
	Convergence (Specificity = 0.82),	Acceptable	
	NPC (Specificity = 0.69),	< Acceptable	
	VMS (Specificity = 0.87),	Acceptable	
	Horz VOR (Specificity = 0.86),	Acceptable	
	Vert VOR (Specificity = 0.87).	Acceptable	
	mVOMs (Specificity = 0.83),	Acceptable	
	Scaled mVOMs (Specificity = 0.83)	Acceptable	
	Total change score (Specificity = 0.82)	Acceptable	
	VOMS Total Cutoff ≥ 4 (Specificity = 0.80)	Acceptable	
	mVOMS Total Cutoff ≥ 4 (Specificity = 0.83)	Acceptable	

Citation	Receiver-Operator Curve (ROC)	Classification	Reference Standard Met?
Mucha et al. 2014	**Receiver-Operator Curve (SRC vs Control) (NPC Cutoff = 5cm, Provocation ≥ 2):**		
	Horz VOR score, VMS scores and NPC distance (AUC = 0.89)	Excellent	☑
	Smooth pursuit (AUC = 0.64)	Fair	
	Horizontal saccade (AUC = 0.68)	Fair	
	Vertical saccade (AUC = 0.65)	Fair	
	Convergence (AUC = 0.64)	Fair	
	Horizontal vestibular ocular reflex (AUC = 0.78)	Acceptable	
	Visual motion sensitivity (AUC = 0.73)	Acceptable	
	NPC Distance (AUC = 0.73)	Acceptable	
McDevitt et al. 2016	**Receiver-Operator Curve (SRC vs Control):**		
	NPC (AUC)= 0.64 (Cutoff = 3.95)	Fair	
Kontos et al. 2021	**Receiver-Operator Curve (SRC vs Control):**		

	Smooth pursuit (AUC = 0.90; 95% CI = 0.86-0.93) (Cutoff = 1)	Outstanding	☑
	Horizontal saccade (AUC = 0.90; 95% CI = 0.86-0.93) (Cutoff = 1)	Outstanding	☑
	Vertical saccade (AUC = 0.90; 95% CI = 0.87-0.93) (Cutoff = 1)	Outstanding	☑
	Near point of convergence (AUC = 0.90; 95% CI = 0.87-0.94) (Cutoff = 1)	Outstanding	☑
	Near point of convergence, cm (AUC = 0.51; 95% CI = 0.46-0.57) (Cutoff = 4.5)	Fair	
	VOR: horizontal (AUC = 0.90; 95% CI = 0.86-0.93) (Cutoff = 2)	Outstanding	☑
	VOR: vertical (AUC = 0.90; 95% CI = 0.87-0.93) (Cutoff = 1)	Outstanding	☑
	Visual-motion sensitivity (AUC = 0.89; 95% CI = 0.86-0.92) (Cutoff = 1)	Excellent	☑
	Total VOMs (AUC = 0.91; 95% CI = 0.88-0.94) (Cutoff = 8)	Outstanding	☑
Ferris et al. 2021a	**Receiver-Operator Curve (NPC Cutoff = 5cm, Provocation ≥ 2)**		
	Total VOMs Excluding NPC Distance (AUC = 0.82)	Excellent	☑
Ferris et al. 2021b	**Receiver-Operator Curve (SRC vs Control):**		
	VOMs Total (AUC = 0.85)	Excellent	☑
	VOMs Total Preseason Change (AUC = 0.75)	Acceptable	
	NPC Distance (AUC = 0.53)	Fair	
	NPC Preseason Change (AUC = 0.58)	Fair	
Knell et al. 2021	**Receiver Operator Curve (Normal vs Protracted Recovery)**		
	Females (AUC = 0.56)	Fair	
	Males (AUC = 0.66)	Fair	
Elbin et al. 2022	**Receiver-Operator Curve (SRC vs Control):**		
	Smooth pursuits (AUC = 0.55) (Cutoff = 1)	Fair	
	Horizontal saccades (AUC = 0.60) (Cutoff = 1)	Fair	
	Vertical saccades (AUC = 0.63) (Cutoff = 1)	Fair	
	NPC symptoms = (AUC = 0.62) (Cutoff = 1)	Fair	
	Horizontal VOR = 0.67 (AUC = 0.67) (Cutoff = 1)	Fair	
	Vertical VOR = (AUC = 0.66) (Cutoff = 1)	Fair	
	VMS (AUC = 0.71) (Cutoff = 1)	Acceptable	
	NPC distance (AUC = 0.58) (Cutoff ≥ 3)	Fair	
	Change (AUC = 0.73) (Cutoff ≥ 3)	Acceptable	
Ferris et al. 2022	**Receiver-Operator Curve (SRC vs Control) (Various Cutoffs):**		
	VOMs total (AUC = 0.85)	Excellent	☑
	Pretest total (AUC = 0.85)	Excellent	☑
	Smooth Pursuit (AUC = 0.85)	Excellent	☑
	Horz Sacc (AUC = 0.85)	Excellent	☑

	Vert Sacc (AUC = 0.85)	Excellent	☑
	Convergence (AUC = 0.85)	Excellent	☑
	NPC Distance (AUC = 0.64)	Fair	
	VMS (AUC = 0.84)	Excellent	☑
	Horz VOR (AUC= 0.84)	Excellent	☑
	Vert VOR (AUC = 0.85)	Excellent	☑
	mVOMs (AUC = 0.85)	Excellent	☑
	Scaled mVOMs (AUC = 0.85)	Excellent	☑
	Total change score (AUC= 0.85)	Excellent	☑
	Peak AUC of any combination (AUC = 0.85)	Excellent	☑
	VOMS Total Cutoff ≥ 4 (AUC = 0.80)	Excellent	☑
	mVOMS Total Cutoff ≥ 4 (AUC = 0.80)	Excellent	☑
	VOMS Overall Cutoff ≥ 8 (AUC = 0.80)	Excellent	☑

Supplementary Table S2.3: Summary Extraction of Alternative Ocular Tools and Technologies Studies.

Citation	DVA	Classification	Reference Standard Met?
Scherer et al. 2013	**Between Session Reliability (24 Hours)**		
	**Yaw:**		
	Active Left (ICC = 0.02; 95% CI = 0.42-0.45)	Poor	
	Active Right (ICC = 0.14; 95% CI = 0.31-0.54)	Poor-Moderate	
	Passive Left (ICC = 0.54; 95% CI = 0.14-0.79)	Poor-Good	
	Passive Right (ICC = 0.41; 95% CI = 0.03-0.72).	Poor-Moderate	
	**Pitch:**		
	Active Down (ICC = 0.30; 95% CI = 0.15-0.65)	Poor-Moderate	
	Active Up (ICC = 0.33; 95% CI = 0.13-0.66)	Poor-Moderate	
	Passive Down (ICC = 0.52; 95% CI = 0.12-0.78)	Poor-Good	
	Passive Up (ICC = 0.61; 95% CI = 0.25-0.83)	Poor-Good	
Kaufman et al. 2013	**Between Session Reliability**		
	**Yaw:**		
	Overall Yaw (ICC = 0.77; 95% CI = 0.60-0.86)	Moderate-Good	☑
	High School Yaw (ICC = 0.72; 95% CI = 0.29-0.89)	Poor-Good	
	University Yaw (ICC = 0.79; 95% CI = 0.55-0.90)	Moderate-Excellent	☑
	**Pitch:**		
	Overall Pitch (ICC = 0.73; 95% CI = 0.52-0.84)	Moderate-Good	
	High School Pitch (ICC = 0.76; 95% CI = 0.0.38-0.90)	Poor-Excellent	☑
	University Pitch (ICC = 0.68; 95% CI = 0.32-0.85)	Poor-Good	
Patterson et al. 2017	**Same Day Reliability (Three Time Points):**		
	Average DVA (ICC = 0.71; 95% CI = 0.45–0.86)	Poor-Good	
	Right DVA (ICC = 0.53; 95% CI = 0.11–0.77)	Poor-Good	
	Left DVA (ICC = 0.48; 95% CI = 0.02–0.74)	Poor-Moderate	
Feller et al. 2021	**Receiver Operator Curve (SRC vs Control):**		
	DVA 2 Reaction Time (ms) (AUC = 0.72; 95% CI = 0.61-0.84)	Acceptable	
	DVA 2 Speed (mph) (AUC = 0.70)	Acceptable	
	DVA 3 Reaction Time (ms) (AUC = 0.74; 0.62-0.86)	Acceptable	

	DVA Speed (mph) (AUC = 0.60)	Fair	
**Citation**	**ImPACT Visual Motor Speed**	**Classification**	**Reference Standard Met?**
Gardner et al. 2012	**Cutoff Exploration:**		
	Sensitivity at >39.2 = 75.60.	< Acceptable	
	Specificity at >39.2 = 56.90.	< Acceptable	
Brett et al. 2016	**Multi-Year Reliability:**		
	**Intra-Class Correlations**		
	Year 1 (ICC = 0.87; 95% CI = 0.83-0.90)	Good-Excellent	☑
	Year 2 (ICC = 0.83; 95% CI = 0.81-0.85)	Good	☑
	Year 3 (ICC = 0.90; 95% CI = 0.85-0.93).	Good-Excellent	☑
	**Pearsons Correlations**		
	Year 1 (r = 0.76)	High	
	Year 2 (r = 0.71)	High	
	Year 3 (r = 0.81)	High	☑
Tsushima et al. 2016	**Two Year Reliability:**		
	Study Duration (ICC = 0.72; 95% CI = 0.65-0.78)	Moderate-Good	
Nelson et al. 2016	**Between Session Reliability:**		
	**Intra-Class Correlations:**		
	7 days (ICC = 0.75)	Moderate	☑
	14 days (ICC = 0.74)	Moderate	
	30 days (ICC = 0.65)	Moderate	
	44 days (ICC = 0.78)	Good	☑
	198 days (ICC = 0.73)	Moderate	
	**Pearsons Correlations:**		
	7 days (r = 0.75)	High	☑
	14 days (r = 0.75)	High	☑
	30 days (r = 0.66)	Moderate	
	44 days (r = 0.78)	High	☑

	198 days (r = 0.75)	High	✓
	**Receiver Operator Curve (SRC vs Control):**		
	Baseline (AUC = 0.58)	Fair	
	24 hours (AUC = 0.71)	Acceptable	
	8 days (AUC = 0.58)	Fair	
	15 days (AUC = 0.59)	Fair	
	45 days (AUC = 0.57)	Fair	
	**Receiver Operator Curve (Prediction of 30-90 Day Recovery):**		
Sufrinko et al. 2017	(AUC = 0.72; 95% CI = 0.57-0.87)	Acceptable	
	*Not significant in 15-29 day recovery		
	**Reliability:**		
Broglio et al. 2018	Year 1-2 (ICC = 0.72; 95% CI = 0.70–0.74)	Moderate	
	Year 1-3 (ICC = 0.66; 95% CI = 0.61–0.71).	Moderate	
	**Between Year Reliability:**		
	Consecutive Years (ICC = 0.85)	Good	✓
	Consecutive Years (r = 0.73)	High	
	**Receiver Operator Curve (SRC vs Control):**		
Ferris et al. 2021b	Raw Data (AUC = 0.51)	Fair	
	Raw Data (Sensitivity = 0.51)	< Acceptable	
	Raw Data (Specificity = 0.51)	< Acceptable	
	Raw vs Pre-Season (AUC = 0.48)	Poor	
	Raw vs Pre-Season (Sensitivity = 0.23)	< Acceptable	
	Raw vs Pre-Season (Specificity = 0.57)	< Acceptable	

Citation	Saccades and Smooth Pursuit Technologies:	Classification	Reference Standard Met?
	**Reliability of Neuro Otologic Test Across Multiple Time Points:**		
	**Horz random task saccade:**		
Cochrane et al. 2019	Primary accuracy (ICC = 0.12; 95% CI = 0.01–0.72)	Poor-Moderate	
	Final accuracy (ICC = 0.20; 95% CI = 0.04–0.60)	Poor-Moderate	
	Latency (ICC = 0.74; 95% CI = 0.59–0.85)	Moderate-Good	

**Vert random task saccade**	
Primary accuracy (ICC = 0.48; 95% CI = 0.26–0.70)	Poor-Moderate
Final accuracy (ICC = 0.03; 95% CI = 0.00–1.00)	Poor-Excellent
Latency (ICC = 0.68; 95% CI = 0.50–0.82)	Moderate-Good
**Horizontal Smooth Pursuit:**	
0.1-Hz velocity gain (ICC = 0.09; 95% CI = 0.00–0.78)	Poor-Good
0.1-Hz position gain (ICC = 0.12; 95% CI = 0.01–0.69)	Poor-Moderate
0.2-Hz velocity gain (ICC = 0.27; 95% CI = 0.08–0.61)	Poor-Moderate
0.2-Hz position gain (ICC = 0.08; 95% CI = 0.00–0.82)	Poor-Good
0.4-Hz velocity gain (ICC = 0.13; 95% CI = 0.01–0.67)	Poor-Moderate
0.4-Hz position gain (ICC = 0.01; 95% CI = 0.00–1.00)	Poor-Excellent
1.0-Hz velocity gain (ICC = 0.70; 95% CI = 0.51–0.83)	Moderate-Good
1.0-Hz position gain (ICC = 0.71; 95% CI = 0.53–0.84)	Moderate-Good
**Vertical Smooth Pursuit:**	
0.1-Hz velocity gain(ICC = 0.56; 95% CI = 0.35–0.75)	Poor-Good
0.1-Hz position gain (ICC = 0.08; 95% CI = 0.00–0.85)	Poor-Good
0.2-Hz velocity gain (ICC = 0.67; 95% CI = 0.49–0.81)	Poor-Good
0.2-Hz position gain (ICC = 0.37; 95% CI = 0.16–0.64)	Poor-Moderate
0.4-Hz velocity gain (ICC = 0.52; 95% CI = 0.31–0.72)	Poor-Moderate
0.4-Hz position gain (ICC = 0.02; 95% CI = 0.00–1.00)	Poor-Excellent
0.75-Hz velocity gain (ICC = 0.51; 95% CI = 0.29–0.73)	Poor-Moderate
0.75-Hz position gain (ICC = 0.37; 95% CI = 0.17–0.64)	Poor-Moderate
**Optokinetic gain:**	
20°/s ccw (ICC = 0.57; 95% CI = 0.36–0.76)	Poor-Moderate
60°/s ccw (ICC = 0.75; 95% CI = 0.59–0.86)	Moderate-Good
**Reliability Pre-Post Practice of Eye-SYNC:**	

	**Pre-Practice:**		
Sundaram et al. 2019	SD Tangential Error (ICC = 0.86; 95% CI = 0.82-0.90)	Good-Excellent	☑
	SD Radial Error (ICC = 0.78; 95% CI = 0.71-0.84)	Moderate-Good	☑
	Phase Error (ICC = 0.83; 95% CI = 0.77-0.87)	Good	☑
	Horizontal Gain (ICC = 0.79; 95% CI = 0.72-0.84)	Moderate-Good	☑
	Vertical Gain (ICC = 0.84; 95% CI = 0.79-0.88)	Good	☑
	**Reliability Pre-Post Practice of Eye-SYNC:**		
	**Post Practice:**		
Sundaram et al. 2019	SD Tangential Error (ICC = 0.88; 95% CI = 0.84-0.91)	Good-Excellent	☑
	SD Radial Error (ICC = 0.91; 95% CI = 0.87-0.91)	Good-Excellent	☑
	Phase Error (ICC = 0.88; 95% CI = 0.84-0.91)	Good-Excellent	☑
	Horizontal Gain (ICC = 0.80; 95% CI = 0.74-0.85)	Moderate-Good	☑
	Vertical Gain (ICC = 0.87; 95% CI = 0.82-0.90)	Good-Excellent	☑
Aloosh et al. 2020	**Reliability of Proprietary Algorithm:**		
	Saccades (ICC = 0.61; 95% CI = 0.20-0.84)	Poor-Good	
	**Between Session Reliability of SMI Red250mobile:**		
	**Adult:**		
Sneigreva et al. 2021	SPS 10° Saccade Count (ICC = 0.86; 95% CI = 0.77–0.91)	Good-Excellent	☑
	Antisaccades (ICC = 0.68; 95% CI = 0.51–0.79)	Moderate-Good	
	SPS 20°saccade count (ICC = 0.60; 955 CI = 0.41–0.74)	Poor-Moderate	
	Fixation Stability (ICC = 0.57; 95% CI = 0.37–0.72)	Poor-Moderate	
	SPS 10° Blink rate (ICC = 0.56; 95% CI = 0.35–0.71).	Poor-Moderate	
	SP saw tooth average blink duration (ICC = 0.53; 95% CI = 0.27–0.71)	Poor-Moderate	
	SPS 10° Saccade Count (r=0.27)	Negligible	
	Antisaccades (r = 0.41)	Low	
	SPS 20°saccade count (r = -0.33)	Low	
	Fixation Stability (ϱ = 0.76).	High	
	SPS 10° Blink rate (ϱ = 0.41).	Low	
	SP saw tooth average blink duration (r = 0.64).	Moderate	
	**Youth:**		
	MGS fast (ICC = 0.99; 95% CI = 0.98–0.99)	Excellent	☑

	Antisaccades (ICC = 0.78; 95% CI = 0.69–0.85)	Moderate-Good	☑
	SP Diagonal Gain (ICC = 0.77; 95% CI = 0.68–0.84)	Moderate-Good	☑
	SP Sinusoidal average blink duration (ICC = 0.63; 95% CI = 0.49–0.74)	Poor-Moderate	
	SP Diagonal blink rate (ICC = 0.62; 95% CI = 0.48–0.73)	Poor-Moderate	
	SP Sinusoidal Gain (ICC = 0.59; 95% CI = 0.45–0.70)	Poor-Moderate	
	SP Sinusoidal blink rate (ICC = 0.55; 95% CI = 0.40–0.67)	Poor-Moderate	
	SP Saw tooth (ICC = 0.54; 95% CI = 0.39–0.66)	Poor-Moderate	
	Fixational stability dispersion average (ICC = 0.52; 95% CI = 0.36–0.65)	Poor-Moderate	
	MGS slow saccade count per step (ICC = 0.52; 95% CI = 0.36–0.65)	Poor-Moderate	
	Antisaccade latency (ICC = 0.51; 95% CI 0.35–0.64)	Poor-Moderate	
	MGS fast (ϱ = 0.61)	Moderate	
	Antisaccades (r = 0.69).	Moderate	
	SP Diagonal Gain (ϱ = 0.08).	Negligible	
	SP Sinusoidal average blink duration (ϱ = 0.36)	Low	
	SP Diagonal blink rate (ϱ = 0.33)	Low	
	SP Sinusoidal Gain (ϱ = 0.61)	Moderate	
	SP Sinusoidal blink rate (ϱ = 0.67)	Moderate	
	SP Saw tooth (ϱ = 0.38)	Low	
	Fixational stability dispersion average (ϱ = 0.51)	Moderate	
	MGS slow saccade count per step (ϱ = 0.33)	Low	
	Antisaccade latency (ϱ = 0.41)	Low	
	**Internal Consistency of SMI Red250 Mobile:**		
	Adult Range (α = 0.54-0.62)	Poor-Questionable	
	Youth Range (α = 0.41-0.61)	Unacceptable-Questionable	
	**Between Session Reliability (Median = 427 Days) of EYE SYNC:**		
	Tangential Variability (ICC = 0.70; 95% CI = 0.50 to 0.83)	Moderate-Good	
Harmon et al. 2021	Radial Variability (ICC = 0.47; 95% CI = 0.19-0.69).	Poor-Moderate	
	**Receiver Operator Curve (SRC vs Control):**		

	Tangential Increase (Sensitivity = 0.48)	< Acceptable	
	Tangential Increase (Specificity = 0.58)	< Acceptable	
	Tangential Variability (AUC = 0.41; 95% CI = 0.30-0.54)	Poor	
	Radial Increase (Sensitivity = 0.52)	< Acceptable	
	Radial Increase (Specificity = 0.61)	< Acceptable	
	Radial Variability (AUC = 0.47 (0.34-0.59)	Poor	

Citation	Additional Tools and Technologies	Classification	Reference Standard Met?
Kaufman et al. 2013	**Reliability of Gaze Stability:**		
	Overall Yaw (ICC = 0.63; 95% CI = 0.36-0.79)	Poor-Good	
	High School Yaw (ICC = 0.75; 95% CI = 0.36-0.90)	Poor-Excellent	✓
	University Yaw (ICC = 0.55; 95% CI = 0.06-0.79)	Poor-Good	
	Overall Pitch (ICC = 0.41; 95% CI = -0.04-0.67)	Poor-Moderate	
	High School Pitch (ICC = 0.53; 95% CI = -0.19-0.81)	Poor-Good	
	University Pitch (ICC = 0.28; 95% CI = -0.52-0.66)	Moderate	
McDevitt et al. 2016	**Receiver Operator Curve (SRC vs Control) for Gaze Stability and Optokinetic Stimulation:**		
	Gaze Stability Signs and Symptoms (AUC = 0.74) (Cutoff = 0.50)	Acceptable	
	Optokinetic Stimulation Signs and Symptoms (AUC = 0.83) (Cutoff = 0.50)	Excellent	✓
	OKS Signs and Symptoms + NPC (AUC = 0.94) NPC Cutoff = 3.95)	Outstanding	✓
Master et al. 2020	**Receiver Operator Curve (SRC vs Control) for Pupillary Light Metrics:**		
	Pupil diameter, mm:		
	Maximum (AUC = 0.78; 95% CI = 0.72 to 0.84)	Acceptable	
	Minimum (AUC = 0.73; 95% CI = 0.67 to 0.80)	Acceptable	
	% Constriction (AUC = 0.74; 95% CI = 0.67 to 0.80)	Acceptable	
	Latency, ms (AUC = 0.50; 95% CI = 0.43 to 0.58)	Poor	
	Constriction velocity, mm/s:		
	Average (AUC = 0.76; 95% CI = 0.70 to 0.82)	Acceptable	
	Peak (AUC = 0.78; 95% CI = 0.72 to 0.84)	Acceptable	

	Dilation velocity, mm/s:	
	Average (AUC = 0.60; 95% CI = 0.52 to 0.67)	Fair
	Peak (AUC = 0.66; 95% CI = 0.59 to 0.73)	Fair
	T75 (s) (AUC = 0.65; 95% CI = 0.58 to 0.72)	Fair
	**Between Session Reliability (Mean = 9.40 ± 6.70 days) of Eyelink 1000:**	
	Box score (ϱ = 0.53)	Moderate
	Left eye skew: right box (ϱ = 0.04)	Negligible
	Left eye skew normalized: right box (ϱ = 0.01)	Negligible
	Right eye skew: right box (ϱ = 0.17)	Negligible
	Right eye skew normalized: right box (ϱ = 0.09)	Negligible
Howell et al. 2018	L/R eye movement variance ratio X: top and bottom box (ϱ = 0.50)	Moderate
	L/R eye movement variance ratio Y: top and bottom box (ϱ = 0.45)	Low
	Left eye distance: bottom box (ϱ = 0.41)	Low
	Left eye distance: left box (ϱ = 0.61)	Moderate
	Left eye movement variance: top box (ϱ = 0.26)	Negligible
	Right eye distance: bottom box (ϱ = 0.29)	Negligible
	Right eye distance: right box (ϱ = 0.34)	Low
	L/R eye movement variance ratio X: bottom box (ϱ = 0.57)	Moderate
	**Receiver Operator Curve (SRC vs Control) for Visio-Vestibular Assessment**	
	**≤10 VVE Reps**	
	Horizontal Saccades (Sensitivity = 0.25)	< Acceptable
	Vertical Saccades (Sensitivity = 0.32)	< Acceptable
	Horizontal Gaze Stability (Sensitivity = 0.19)	< Acceptable
Storey et al. 2022	Vertical Gaze Stability (Sensitivity = 0.23)	< Acceptable
	Optimal Cutoff of 1 at ≤10 Reps (Sensitivity = 0.39)	< Acceptable
	**≤20 VVE Reps**	
	Horizontal Saccades (Sensitivity = 0.50)	< Acceptable
	Vertical Saccades (Sensitivity = 0.32)	< Acceptable

	Horizontal Gaze Stability (Sensitivity = 0.19)	< Acceptable
	Vertical Gaze Stability (Sensitivity = 0.23)	< Acceptable
	Optimal Cutoff of 1 at ≤20 Reps (Sensitivity = 0.59)	< Acceptable
	**≤30 VVE Reps**	
	Horizontal Saccades (Sensitivity = 0.69)	< Acceptable
	Vertical Saccades (Sensitivity = 0.74)	< Acceptable
	Horizontal Gaze Stability (Sensitivity = 0.71)	< Acceptable
	Vertical Gaze Stability (Sensitivity = 0.72)	< Acceptable
	Optimal Cutoff of 3 at ≤30 Reps (Sensitivity = 0.64)	< Acceptable
	Optimal Cutoff of 4 at ≤30 Reps (Sensitivity = 0.55)	< Acceptable
	**≤10 VVE Reps**	
	Horizontal Saccades (Specificity = 1.00)	Good-Excellent
	Vertical Saccades (Specificity = 0.99)	Good-Excellent
	Horizontal Gaze Stability (Specificity = 1.00)	Good-Excellent
	Vertical Gaze Stability (Specificity = 1.00)	Good-Excellent
	Optimal Cutoff of 1 at ≤10 Reps (Specificity = 0.99)	Good-Excellent
	**≤20 VVE Reps**	
	Horizontal Saccades (Specificity = 0.99)	Good-Excellent
Storey et al. 2022	Vertical Saccades (Specificity = 0.99)	Good-Excellent
	Horizontal Gaze Stability (Specificity = 1.00)	Good-Excellent
	Vertical Gaze Stability (Specificity = 1.00)	Good-Excellent
	Optimal Cutoff of 1 (Specificity = 0.96)	Good-Excellent
	**≤30 VVE Reps**	
	Horizontal Saccades (Specificity = 0.52)	< Acceptable
	Vertical Saccades (Specificity = 0.54)	< Acceptable
	Horizontal Gaze Stability (Specificity = 0.74)	< Acceptable
	Vertical Gaze Stability (Specificity = 0.76)	< Acceptable
	Optimal Cutoff of 3 (Specificity = 0.84)	Acceptable

Optimal Cutoff of 4 (Specificity = 0.90)	Good-Excellent
**Receiver-Operator Curve (SRC vs Control):**	
≤10 Reps (AUC = 0.69; 95% CI = 0.63-0.75)	Fair
≤20 Reps (AUC = 0.79 ; 95% CI = 0.72-0.85)	Acceptable
≤30 Reps (AUC = 0.76; 95% CI 0.68-0.84)	Acceptable
