# Supplementary material for: Clinical Utility of Ocular Assessments in Sport-Related Concussion: A Scoping Review
Source: J Funct Morphol Kinesiol. 2024 Sep 4;9(3):157. doi: 10.3390/jfmk9030157 (PMC11417888; doi:10.3390/jfmk9030157)
Supplement: Supplementary file 1 [file jfmk-09-00157-s001.zip › Supplementary File S3.pdf]

Table S3.1: Risk of bias analysis for included diagnostic accuracy studies using the revised QUADAS-2. Green = low, red = high, orange = unclear (for both risk of bias and applicability).

Study	Diagnostic Accuracy						
	Risk of Bias				Applicability Concerns		
	Patient	Index	Reference	Flow	Patient	Index	Reference
Gardener et al. 2012	Green	Red	Green	Green	Green	Green	Green
Mucha et al. 2014	Green	Red	Green	Red	Green	Green	Green
King et al. 2015a	Red	Green	Green	Green	Green	Green	Green
King et al. 2015b	Red	Red	Green	Green	Green	Green	Green
Galetta et al. 2015	Red	Red	Red	Green	Green	Green	Green
McDevitt et al. 2016	Red	Red	Green	Green	Green	Green	Green
Nelson et al. 2016	Red	Red	Orange	Green	Green	Green	Green
Guzowski et al. 2017	Orange	Orange	Orange	Orange	Green	Green	Orange
Sufrinko et al. 2017	Green	Orange	Orange	Green	Green	Green	Green
Dhawan et al. 2017	Red	Green	Green	Green	Green	Green	Green
Molloy et al. 2017	Red	Green	Orange	Green	Green	Green	Green
DuPrey et al. 2017	Red	Orange	Green	Green	Green	Green	Green
Hecimovich et al. 2018a	Orange	Orange	Red	Green	Green	Green	Red
Hecimovich et al. 2018b	Red	Red	Green	Green	Green	Green	Green
Naidu et al. 2018	Red	Orange	Orange	Green	Green	Green	Green
Fuller et al. 2019	Red	Red	Green	Green	Green	Green	Green
Master et al. 2020	Red	Red	Green	Red	Green	Green	Green
Harmon et al. 2021	Red	Orange	Red	Green	Green	Green	Green
Feller et al. 2021	Red	Red	Green	Red	Green	Green	Green
Ferris et al. 2021a	Red	Orange	Green	Green	Green	Green	Green
Ferris et al. 2021b	Red	Orange	Orange	Green	Green	Green	Green
Knell et al. 2021	Red	Red	Red	Green	Green	Green	Green
Kontos et al. 2021	Red	Red	Green	Green	Green	Green	Green
Elbin et al. 2022	Red	Red	Green	Red	Green	Green	Green
Ferris et al. 2022	Red	Orange	Green	Green	Green	Green	Green
Hecimovich et al. 2022	Red	Red	Green	Green	Green	Green	Green
Storey et al. 2022	Red	Orange	Green	Red	Green	Green	Green
Le et al. 2023	Red	Red	Green	Green	Green	Green	Green

Table S3.2: Risk of bias analysis for included reliability studies using the COSMIN reliability criteria. Dark green = very good, light green = adequate, orange = doubtful, red = inadequate, N/A = not applicable.

	Reliability					
**Study**	**Stability**	**Interval**	**Conditions**	**Flaws**	**ICC**	**Kappa**
Galetta et al. 2011b						N/A
Scherer et al. 2013						N/A
Leong et al. 2013	N/A					N/A
Kaufman et al. 2013						N/A
Yevseyenkov et al. 2013						N/A
Leong et al. 2013						N/A
Leong et al. 2015						N/A
King et al. 2015a						N/A
King et al. 2015b						
Vartiainen et al. 2015						N/A
Pearce et al. 2015						N/A
Kawata et al. 2015	N/A					N/A
Walsh et al. 2016						N/A
Brett et al. 2016						N/A
Alsalaheen et al. 2016						N/A
Smolyansky et al. 2016						N/A
Tsushima et al. 2016						N/A
Nelson et al. 2016	N/A					N/A
Kawata et al. 2016	N/A					N/A
Patterson et al. 2017	N/A					N/A
Oberlander et al. 2017						N/A
Weise et al. 2017						N/A
Broglio et al. 2018						

Table S3.3: Risk of bias analysis for included reliability studies using the COSMIN reliability criteria continued. Dark green = very good, light green = adequate, orange = doubtful, red = inadequate, N/A = not applicable.

	Reliability					
**Study**	**Stability**	**Interval**	**Conditions**	**Flaws**	**ICC**	**Kappa**
Hecimovich et al. 2018b	N/A					
Moran and Covassin et al. 2018a						N/A
Howell et al. 2018						N/A
Naidu et al. 2018	N/A					N/A
Worts et al. 2018	N/A					N/A
Zonner et al. 2018						N/A
Cochrane et al. 2019						N/A
Sundaram et al. 2019	N/A					N/A
Breedlove et al. 2019						N/A
Elbin et al. 2019						N/A
White-Schwoch et al. 2019						N/A
Buttner et al. 2020	N/A					
King et al. 2020						N/A
Aloosh et al. 2020						N/A
Kontos et al. 2020						N/A
Heick et al. 2021						N/A
Ferris et al. 2021b						
Sneigreva et al. 2021						N/A
Harmon et al. 2021						N/A
De Rossi 2022						N/A
Kalbfell et al. 2023						N/A
Zuidema et al. 2023						N/A
Moran et al. 2023						

Table S3.4: Risk of bias analysis for included internal consistency studies using the COSMIN criteria. Dark green = very good, orange = doubtful, red = inadequate.

Internal Consistency		
**Study**	**Calculation**	**Scoring**
Galetta et al. 2011a		
Galetta et al. 2011b		
King et al. 2012		
King et al. 2013		
Mucha et al. 2014		
Kontos et al. 2016		
Moran and Covassin 2018a		
Moran and Covassin 2018b		
Iverson et al. 2019		
Snegireva et al. 2021		
Worts et al. 2020		
Kontos et al. 2020		
Moran et al. 2023		
Anderson et al. 2024		
